# Efficient Construction of a Complete Index for Pan-Genomics Read Alignment

**DOI:** 10.1089/cmb.2019.0309

**Published:** 2020-04-08

**Authors:** Alan Kuhnle, Taher Mun, Christina Boucher, Travis Gagie, Ben Langmead, Giovanni Manzini

**Affiliations:** ^1^ Department of Computer Science, Florida State University, Tallahassee, Florida.; ^2^ Department of Computer and Information Science and Engineering, University of Florida, Gainesville, Florida.; ^3^ Department of Computer Science, John Hopkins University, Baltimore, Maryland.; ^4^ Faculty of Computer Science, Dalhousie University, Halifax, Canada.; ^5^ School of Computer Science and Telecommunications, Universidad Diego Portales and CeBiB, Santiago, Chile.; ^6^ Department of Science and Technological Innovation, University of Eastern Piedmont, Alessandria, Italy.

**Keywords:** Burrows–Wheeler Transform, indexing, *r*-index, pan-genomics

## Abstract

Short-read aligners predominantly use the FM-index, which is easily able to index one or a few human genomes. However, it does not scale well to indexing collections of thousands of genomes. Driving this issue are the two chief components of the index: (1) a rank data structure over the Burrows–Wheeler Transform (BWT) of the string that will allow us to find the interval in the string's suffix array (SA), and (2) a sample of the SA that—when used with the rank data structure—allows us to access the SA. The rank data structure can be kept small even for large genomic databases, by run-length compressing the BWT, but until recently there was no means known to keep the SA sample small without greatly slowing down access to the SA. Now that (SODA 2018) has defined an SA sample that takes about the same space as the run-length compressed BWT, we have the design for efficient FM-indexes of genomic databases but are faced with the problem of building them. In 2018, we showed how to build the BWT of large genomic databases efficiently (WABI 2018), but the problem of building the sample efficiently was left open. We compare our approach to state-of-the-art methods for constructing the SA sample, and demonstrate that it is the fastest and most space-efficient method on highly repetitive genomic databases. Lastly, we apply our method for indexing partial and whole human genomes and show that it improves over the FM-index-based Bowtie method with respect to both memory and time and over the hybrid index-based CHIC method with respect to query time and memory required for indexing.

## 1. Introduction

The FM-index, which is a compressed subsequence index based on Burrows**–**Wheeler Transform (BWT), is the primary data structure of the majority of short-read aligners—including Bowtie (Langmead et al., 2008), BWA (Li and Durbin, 2009), and SOAP2 (Li et al., 2009). These aligners build an FM-index-based data structure of sequences from a given genomic database and then use the index to perform queries that find approximate matches of sequences to the database. While these methods can easily index one or a few human genomes, they do not scale well to thousands of genomes. This is problematic in analysis of the data produced by consortium projects, which routinely have several 1000 genomes.

In this article, we address this need by introducing and implementing an algorithm for efficiently constructing the FM-index, which allows for the FM-index construction to scale to larger sets of genomes. To understand the challenge and solution behind our method, consider the two principal components of the FM-index: first, a rank data structure over the BWT of the string that enables us to find the interval in the string's suffix array (SA) containing pointers to starting positions of occurrences of a given pattern (and to compute how many such occurrences there are); second, a sample of the SA that, when used with the rank data structure, allows us to access the SA (so we can list those starting positions). Searching with an FM-index can be summarized as follows: starting with the empty suffix, for each proper suffix of the given pattern, we use rank queries at the end of the BWT interval containing the characters immediately preceding occurrences of that suffix in the string, to compute the interval containing the characters immediately preceding occurrences of the suffix of length 1 greater; when we have the interval containing the characters immediately preceding occurrences of the whole pattern, we use an SA sample to list the contexts of the corresponding interval in the SA, which are the locations of those occurrences.

Although it is possible to use a compressed implementation of the rank data structure that does not become much slower or larger even for thousands of genomes, the same cannot be said for the SA sample. The product of the size and the access time must be at least linear in the length of the string for the standard SA sample. This implies that the FM-index will become much slower and/or much larger as the number of genomes in the databases grows significantly. This bottleneck has forced researchers to consider variations of FM-indexes adapted for massive genomic data sets, such as the Valenzuela et al. (2018) pan-genomic index or the Garrison et al. (2018) variation graph. Some of these proposals use elements of the FM-index, but all deviate in substantial ways from the description above. Not only does this mean they lack the FM-index's long and successful track record, it also means they usually do not give us the BWT intervals for all the suffixes as we search (whose lengths are the suffixes' frequencies, and thus, a tightening sequence of upper bounds on the whole pattern's frequency), nor even the final interval in the SA (which is an important input in other string processing tasks).

Recently, Gagie et al. (2018) proposed a different approach to SA sampling, which takes space proportional to that of the compressed rank data structure while still allowing reasonable access times. While their result yielded a potentially practical FM-index on massive databases, it did not directly lead to a solution since the problem of how to efficiently construct the BWT and SA sample remained open. In a direction toward fully realizing the theoretical result of Gagie et al. (2018), Boucher et al. (2018) showed how to build the BWT of large genomic databases efficiently. We refer to this construction as *prefix-free parsing*. It takes as input string *S* and in one pass generates a dictionary and a parse of *S* with the property that the BWT can be constructed from dictionary and parse using workspace proportional to their total size and in *O*(|*S*|) time. Yet, the resulting index of Boucher et al. (2018) lacks the SA sample and therefore does not support locating. This makes this index not directly applicable to many bioinformatic applications, such as sequence alignment.

### 1.1. Our contributions

In this article, we present a solution for building the FM-index^*^ for very large data sets by showing that we can build the BWT and Gagie et al.'s (2018) SA sample together in roughly the same time and memory needed to construct the BWT alone. We note that this algorithm is also based on prefix-free parsing. Thus, we begin by describing how to construct the BWT from the prefix-free parse, and then, we show that it can be modified to build the SA sample in addition to the BWT in roughly the same time and space. We implement this approach, and we refer to the resulting implementation as bigbwt. We compare it to state-of-the-art methods for constructing the SA sample and demonstrate that bigbwt is currently the fastest and most space-efficient method for constructing the SA sample on large genomic databases.

Next, we demonstrate the applicability of our method to short-read alignment. In particular, we compare the memory and time needed by our method to build an index for collections of chromosome 19 with those of Bowtie (Langmead et al., 2008) and CHIC (Valenzuela and Mäkinen, 2017). We also compare the sizes of the resulting indexes as well as the amount of time required to perform several locate queries against the indexes. We find that Bowtie is unable to build indexes for our largest collections (500 or more) because it exhausted memory, whereas our method is able to build indexes up to 2000 chromosome 19s (and likely beyond). At 250 chromosome 19 sequences, our method requires only about 2% of the time and 6% the peak memory of Bowtie's. While CHIC can produce the smallest indexes for smaller sequence collections, this comes at the cost of higher indexing memory footprint and dramatically higher query time. Lastly, we demonstrate that it is possible to index collections of whole human genome assemblies with sublinear scaling as the size of the collection grows.

### 1.2. Related work

The development of methods for building the FM-index on large data sets is closely related to the development of short-read aligners for pan-genomics—an area where there is growing interest (Schneeberger et al., 2009; Danek et al., 2014; Gagie and Puglisi, 2015). Here, we briefly describe some previous approaches to this problem and detail their connection to the work in this article. We note that the majority of pan-genomic aligners require building the FM-index for a population of genomes and thus could increase proficiency using the methods described in this article.

GenomeMapper (Schneeberger et al., 2009), the method of Danek et al. (2014), and Generalized Compressed Suffix Array (GCSA) (Sirén et al., 2014) represent the genomes in a population as a graph and then reduce the alignment problem to finding a path within the graph. Hence, these methods require all possible paths to be identified, which is exponential in the worst case. Some of these methods—such as GCSA—use the FM-index to store and query the graph and could capitalize on our approach by building the index in the manner described here. Another set of approaches (Mäkinen et al., 2010; Ferrada et al., 2014; Gagie and Puglisi, 2015; Valenzuela and Mäkinen, 2017) considers the reference pan-genome as the concatenation of individual genomes and exploits redundancy using a compressed index.

The hybrid index (Ferrada et al., 2014) operates on a Lempel-Ziv compression of the reference pan-genome. An input parameter *M* sets the maximum length of reads that can be aligned. This has a major impact on the final size of the index. For this reason, the hybrid index is suitable mainly for short-read alignment, although there have been recent heuristic modifications to allow for longer alignments (Ferrada et al., 2018). In contrast, the *r*-index, of which we provide an implementation in this work, has no such length limitation. The most recent implementation of the hybrid index is CHIC (Valenzuela et al., 2018; based on CHICO; Valenzuela, 2016). Although CHIC has support for counting multiple occurrences of a pattern within a genomic database, it is an expensive operation, namely O(ℓloglogn), where *ℓ* is the number of occurrences in the databases and *n* is the length of the database. However, the *r*-index is capable of counting all occurrences of a pattern of length *m* in *O*(*m*) time up to polylog factors. There are a number of other approaches building off the hybrid index or similar ideas (Wandelt et al., 2013; Danek et al., 2014); for an extended discussion, we refer the reader to the survey of Gagie and Puglisi (2015).

Finally, a third set of approaches (Huang et al., 2013; Maciuca et al., 2016) attempts to encode variants within a single reference genome. BWBBLE by Huang et al. (2013) follows this by supplementing the alphabet to indicate if multiple variants occur at a single location. This approach does not support counting of the number of variants matching a specific alignment; also, it suffers from memory blow-up when larger structural variations occur.

## 2. Background

### 2.1. BWT and FM indexes

Consider a string *S* of length *n* from a totally ordered alphabet Σ, such that the last character of *S* is lexicographically less than any other character in *S*. Let *F* be the list of *S'*s characters sorted lexicographically by the suffixes starting at those characters, and let *L* be the list of *S'*s characters sorted lexicographically by the suffixes starting immediately after those characters. The list *L* is termed the BWT (Burrows and Wheeler, 1994) of *S* and denoted BWT. If S[i] is in position *p* in *F*, then S[i−1] is in position *p* in *L*. Moreover, if S[i]=S[j], then S[i] and S[j] have the same relative order in both lists; otherwise, their relative order in *F* is the same as their lexicographic order. This means that if S[i] is in position *p* in *L* then, assuming arrays are indexed from 0 and ≺ denotes lexicographic precedence, in *F* it is in position *j_i_* = |{*h*: *S*[*h*] ≺ *S*[*i*]}| + |{*h*: *L*[*h*] = *S*[*i*], *h* ≤ *p*}| − 1. The mapping *i*↦*j_i_* is termed the LF mapping. Finally, notice that the last character in *S* always appears first in *L*. By repeated application of the LF mapping, we can invert the BWT, that is, recover *S* from *L*. Formally, the SA of the string *S* is an array such that entry *i* is the starting position in *S* of the *i*th largest suffix in lexicographical order. The above definition of the BWT is equivalent to the following:

(1) BWT[i]=S[(SA[i]−1) modn].

The BWT was introduced as an aid to data compression: it moves characters followed by similar contexts together and thus makes many strings encountered in practice locally homogeneous and easily compressible. Ferragina and Manzini (2000) showed how the BWT may be used for *indexing* a string *S*: given a pattern *P* of length *m* < *n*, find the number and location of all occurrences of *P* within *S*. If we know the range BWT(S)[i..j] occupied by characters immediately preceding occurrences of a pattern *Q* in *S*, then we can compute the range BWT(S)[i′..j′] occupied by characters immediately preceding occurrences of *cQ* in *S*, for any character *c* ∈Σ, since

$$\begin{array}{cc} & i\prime =|\left\{h:S\left[h\right]≺c\right\}|+|\left\{h:S\left[h\right]=c,h<i\right\}|\\ & j\prime =|\left\{h:S\left[h\right]≺c\right\}|+|\left\{h:S\left[h\right]=c,h\leq j\right\}|-1. \end{array}$$

Notice j′−i′+1 is the number of occurrences of *cQ* in *S*. The essential components of an FM-index for *S* are, first, an array storing |{h:S[h]≺c}| for each character *c* and, second, a rank data structure for BWT that quickly tells us how often any given character occurs up to any given position.^†^ To be able to locate the occurrences of patterns in *S* (in addition to just counting them), the FM-index uses a sampled^‡^ SA of *S* and a bit vector indicating the positions in the BWT of the characters preceding the sampled suffixes.

### 2.2. Prefix-free parsing

Next, we give an overview of prefix-free parsing, which produces a dictionary D and a parse P by sliding a window of fixed width through the input string *S* and dividing it into variable-length overlapping substrings with delimiting prefixes and suffixes. We refer the reader to Boucher et al. (2018) for the formal proofs and Section 3.1 for the algorithmic details. A rolling hash function identifies when substrings are parsed into elements of a dictionary, which is a set of substrings of *S*. Intuitively, for a repetitive string, the same dictionary phrases will be encountered frequently.

We now formally define the dictionary D and parse P. Given a string^§^ *S* of length *n*, window size w∈ℕ, and modulus p∈ℕ, we construct the dictionary D of substrings of *S* and the parse P as follows; we let *f* be a hash function on strings of length *w*, and let T be the sequence of substrings *W* = *S*[*s*, *s* + *w* − 1] such that *f* (*W*) ≡ 0(mod *p*) or *W* = *S*[0, *w* − 1] or *W* = *S*[*n* − *w* + 1, *n* − 1], ordered by initial position in *S*; let $T=\left(W_{1}=S\left[s_{1},s_{1}+w-1\right],\ldots ,W_{k}=\left[s_{k},s_{k}+w-1\right]\right)$. By construction, the strings

$$S\left[s_{1},s_{2}+w-1\right],S\left[s_{2},s_{3}+w-1\right],\ldots ,S\left[s_{k-1},s_{k}+w-1\right]$$

form a parsing of *S*, in which each pair of consecutive strings *S*[*s_i_*, *s_i_*_+1_ + *w* − 1] and *S*[*s_i_*_+1_, *s_i_*_+2_ + *w* − 1] overlaps by exactly *w* characters. We define $D=\left\{S\left[s_{i},s_{i+1}+w-1\right]:1\leq i<k\right\}$; that is, D consists of the set of the unique substrings *s* of *S* such that |*s*| > *w* and the first and last *w* characters of *s* form consecutive elements in T. If *S* has many repetitions, we expect that |D|≪k. With a little abuse of notation, we define the parsing P as the sequence of lexicographic ranks of substrings in D: $P={\left({rank}_{D}\left(S\left[s_{i},s_{i+1}+w-1\right]\right)\right)}_{i=1}^{k-1}$. The parse P indicates how *S* may be reconstructed using elements of D. The dictionary D and parse P may be constructed in one pass over *S* in $O\left(n+|D|\log\left|D\right|\right)$ time if the hash function *f* can be computed in constant time.

### 2.3. *r*-index locating

Policriti and Prezza (2018) showed that if we have stored SA[k] for each value *k* such that BWT[k] is the beginning or end of a run (i.e., a maximal nonempty unary substring) in BWT, and we know both the range BWT[i..j] occupied by characters immediately preceding occurrences of a pattern *Q* in *S* and the starting position of one of those occurrences of *Q*, then when we compute the range BWT[i′..j′] occupied by characters immediately preceding occurrences of *cQ* in *S*, we can also compute the starting position of one of those occurrences of *cQ*. Bannai et al. (2018) then showed that even if we have stored only SA[k] for each value *k* such that BWT[k] is the beginning of a run, then as long as we know SA[i], we can compute SA[i′].

Gagie et al. (2018) showed that if we have stored in a predecessor data structure SA[k] for each value *k* such that BWT[k] is the beginning of a run in BWT, with $\phi^{-1}\left(SA\left[k\right]\right)=SA\left[k+1\right]$ stored as satellite data, then given SA[h] we can compute SA[h+1] in O(loglogn) time as $SA\left[h+1\right]=\phi^{-1}\left(pred\left(SA\left[h\right]\right)\right)+SA\left[h\right]-pred\left(SA\left[h\right]\right),$ where *pred*(·) is a query to the predecessor data structure. Combined with Bannai et al.'s (2018) result, this means that while finding the range BWT[i..j] occupied by characters immediately preceding occurrences of a pattern *Q*, we can also find SA[i] and then report SA[i+1..j] in O((j−i)loglogn)-time; that is, O(loglogn)-time per occurrence.

Gagie et al. (2018) gave the name *r*-index to the index resulting from combining a rank data structure over the run-length-compressed BWT with their SA sample, and Bannai et al. (2018) used the same name for their index. Since our index is an implementation of theirs, we keep this name; on the contrary, we do not apply it to indexes based on run-length-compressed BWTs that have standard SA samples or no SA samples at all.

## 3. Methods

Here we describe our algorithm for building the SA or the sampled SA from the prefix-free parse of an input string *S*, which is used to build the *r*-index. We first review the algorithm from Boucher et al. (2018) for building the BWT of *S* from the prefix-free parse. Next, we show how to modify this construction to compute the SA or the sampled SA along with the BWT.

### 3.1. Construction of BWT from prefix-free parse

We assume we are given a prefix-free parse of *S*[1..*n*] with window size *w* consisting of a dictionary D and a parse P. We represent the dictionary as a string $D\left[1..\ell \right]=t_{1}\#t_{2}\#\cdots t_{d-1}\#t_{d}\#$ where *t_i_'*s are the dictionary phrases in lexicographic order and # is a unique separator. We assume we have computed the SA of D, denoted by ${SA}_{D}\left[1..\ell \right]$ in the following, and the BWT of P, denoted ${BWT}_{P}$, and the array Occ[1,d] such that Occ[i] stores the number of occurrences of the dictionary phrase *t_i_* in the parse. These preliminary computations take O(|D|+|P|) time.

By the properties of the prefix-free parsing, each suffix of *S* is prefixed by *exactly one* suffix *α* of a dictionary phrase *t_j_* with |*α*| > *w*. We call *α_i_* the *representative prefix* of the suffix *S*[*i*..*n*]. From the uniqueness of the representative prefix, we can partition *S'*s suffix array *SA*[1..*n*] into *k* ranges

$$\left[b_{1},e_{1}\right],\left[b_{2},e_{2}\right],\left[b_{3},e_{3}\right],\ldots ,\left[b_{k},e_{k}\right]$$

with *b*_1_ = 1, *b_i_* = *e_i_*_−1_ + 1 for *i* = 2, …, *k*, and *e_k_* = *n*, such that for *i* = 1, …, *k* all suffixes

$$S\left[SA\left[b_{i}\right]..n\right],S\left[SA\left[b_{i}+1\right]..n\right],\ldots ,S\left[SA\left[e_{i}\right]..n\right]$$

have the same representative prefix *α_i_*. By construction *α*_1_ ≺ *α*_2_ ≺⋯ ≺ *α_k_*.

By construction, any suffix D[i..ℓ] of the dictionary D is also prefixed by the suffix of a dictionary phrase. For *j* = 1, …, *ℓ*, let *β_j_* denote the longest prefix of $D\left[{SA}_{D}\left[j\right]..\ell \right]$, which is the suffix of a phrase (i.e., $D\left[{SA}_{D}\left[j\right]+|\beta_{j}|\right]=\#$). By construction, the strings *β_j'_*s are lexicographically sorted *β*_1_ ≺ *β*_2_ ≺⋯ ≺ *β_ℓ_*. Clearly, if we compute *β*_1_, …, *β_ℓ_* and discard those such that |*β_j_*| ≤ *w*, the remaining *β_j'_*s will coincide with the representative prefixes *α_i'_*s. Since both *β_j'_*s and *α_i'_*s are lexicographically sorted, this procedure will generate the representative prefixes in the order *α*_1_, *α*_2_, …, *α_k_*. We note that more than one *β_j_* can be equal to some *α_i_* since different dictionary phrases can have the same suffix.

We scan ${SA}_{D}\left[1..\ell \right]$, compute *β*_1_, …*β_ℓ_*, and use these strings to find the representative prefixes. As soon as we generate an *α_i_*, we compute and output the portion $BWT\left[b_{i},e_{i}\right]$ corresponding to the range [*b_i_*, *e_i_*] associated with *α_i_*. To implement the above strategy, assume there are exactly *k* entries in ${SA}_{D}\left[1..\ell \right]$ prefixed by *α_i_*. This means that there are *k* distinct dictionary phrases *t_i_*_1_, *t_i_*_2_, …, *t_ik_* that end with *α_i_*. Hence, the range [*b_i_*, *e_i_*] contains $z_{i}=e_{i}-b_{i}+1=\sum_{h=1}^{k}Occ\left[i_{h}\right]$ elements. To compute $BWT\left[b_{i},e_{i}\right]$ we need to (1) find the symbol immediately preceding each occurrence of *α_i_* in *S*, and (2) find the lexicographic ordering of *S'*s suffixes prefixed by *α_i_*. We consider the latter problem first.

#### 3.1.1. Computing the lexicographic ordering of suffixes

For *j* = 1, …, *z_i_*, consider the *j*th occurrence of *α_i_* in *S* and let *i_j_* denote the position in the parsing of *S* of the phrase ending with the *j*th occurrence of *α_i_*. In other words, $P\left[i_{j}\right]$ is a dictionary phrase ending with *α_i_* and *i*_1_ < *i*_2_ < ⋯ <*i_zi_*. By the properties of ${BWT}_{P}$, the lexicographic ordering of *S'*s suffixes prefixed by *α_i_* coincides with the ordering of the symbols $P\left[i_{j}\right]$ in ${BWT}_{P}$. In other words, $P\left[i_{j}\right]$ precedes $P\left[i_{h}\right]$ in ${BWT}_{P}$ if and only if *S'*s suffix prefixed by the *j*th occurrence of *α_i_* is lexicographically smaller than *S'*s suffix prefixed by the *h*th occurrence of *α_i_*.

We could determine the desired lexicographic ordering by scanning ${BWT}_{P}$ and noticing which entries coincide with one of the dictionary phrases *t_i_*_1_, …, *t_ik_* that end with *α_i_*, but this would clearly be inefficient. Instead, for each dictionary phrase *t_i_*, we maintain an array $IL_{i}$ of length Occ[i] containing the indexes *j* such that ${BWT}_{P}\left[j\right]=i$. These sorts of “inverted lists” are computed at the beginning of the algorithm and replace the ${BWT}_{P}$, which can be discarded.

#### 3.1.2. Finding the symbol preceding *α_i_*

Given a representative prefix *α_i_* from ${SA}_{D}$, we retrieve the indexes *i*_1_, …, *i_k_* of the dictionary phrases *t_i_*_1_, …, *t_ik_* that end with *α_i_*. Then, we retrieve the inverted lists $IL_{i_{1}},\ldots IL_{i_{k}}$ and we merge them, obtaining the list of the *z_i_* positions *y*_1_ < *y*_2_ < ⋯ <*y_zi_* such that ${BWT}_{P}\left[y_{j}\right]$ is a dictionary phrase ending with *α_i_*. Such a list implicitly provides the lexicographic order of *S'*s suffixes starting with *α_i_*.

To compute the BWT, we need to retrieve the symbols preceding such occurrences of *α_i_*. If *α_i_ is not* a dictionary phrase, then *α_i_* is a proper suffix of the phrases *t_i_*_1_, …, *t_ik_* and the symbols preceding *α_i_* in *S* are those preceding *α_i_* in *t_i_*_1_, …, *t_ik_* that we can retrieve from D[1..ℓ] and ${SA}_{D}\left[1..\ell \right]$. If *α_i_ coincides* with a dictionary phrase *t_j_*, then it cannot be a suffix of another phrase. Hence, the symbols preceding *α_i_* in *S* are those preceding *t_j_* in *S* that we store at the beginning of the algorithm in an auxiliary array $PR_{j}$ along with the inverted list $IL_{j}$.

### 3.2. Construction of SA and SA sample along with the BWT

We now show how to modify the above algorithm so that, along with BWT, it computes the full SA of *S* or the sampled SA consisting of the values *SA*[*s*_1_], …, *SA*[*s_r_*] and *SA*[*e*_1_], …, *SA*[*e_r_*], where *r* is the number of maximal nonempty runs in BWT and *s_i_* and *e_i_* are the starting and ending positions in BWT of the *i*th such run, respectively. Note that if we compute the sampled SA, the actual output will consist of *r* start-run pairs $\left\langle s_{i},SA\left[s_{i}\right]\right\rangle$ and *r* end-run pairs $\left\langle e_{i},SA\left[e_{i}\right]\right\rangle$ since the *SA* values alone are not enough for the construction of the *r*-index.

We solve both problems using the following strategy. Simultaneously to each entry BWT[j], we compute the corresponding entry *SA*[*j*]. Then, if we need the sampled SA, we compare BWT[j−1] and BWT[j] and if they differ, we output the pair ⟨j−1,SA[j−1]⟩ among the end-runs and the pair ⟨j,SA[j]⟩ among the start-runs. To compute the SA entries, we only need *d* additional arrays $EP_{1},\ldots EP_{d}$ (one for each dictionary phrase), where $|EP_{i}|=|IL_{i}|=Occ\left[i\right]$, and $EP_{i}\left[j\right]$ contains the ending position in *S* of the dictionary phrase, which is in position $IL_{i}\left[j\right]$ of ${BWT}_{P}$.

Recall that in the above algorithm for each occurrence of a representative prefix *α_i_*, we compute the indexes *i*_1_, …, *i_k_* of the dictionary phrases *t_i_*_1_, …, *t_ik_* that end with *α_i_*. Then, we use the lists $IL_{i_{1}},\ldots ,IL_{i_{k}}$ to retrieve the positions of all the occurrences of *t_i_*_1_, …, *t_ik_* in ${BWT}_{P}$, thus establishing the relative lexicographic order of the occurrences of the dictionary phrases ending with *α_i_*. To compute the corresponding SA entries, we need the starting position in *S* of each occurrence of *α_i_*. Since the ending position in *S* of the phrase with relative lexicographic rank $IL_{i_{h}}\left[j\right]$ is $EP_{i_{h}}\left[j\right]$, the corresponding SA entry is $EP_{i_{h}}\left[j\right]-|\alpha_{i}|+1$. Hence, along with each BWT entry, we obtain the corresponding SA entry, which is saved to the output file if the full SA is needed, or further processed as described above if we need the sampled SA.

## 4. Time and Memory Usage for SA and SA Sample Construction

We compare the running time and memory usage of bigbwt with the following methods, which represent the current state-of-the-art.

### 4.1. bwt2sa

Once the BWT has been computed, the SA or SA sample may be computed by applying the *LF* mapping to invert the BWT and the application of Equation (1). Therefore, as a baseline, we use bigbwt to construct the BWT only, as in Boucher et al. (2018); we use bigbwt since it seems best suited to the inputs we consider. Next, we load the BWT as a Huffman-compressed string with access, rank, and select support to compute the *LF* mapping. We step backward through the BWT and compute the entries of the SA in nonconsecutive order. Finally, these entries are sorted in external memory to produce the SA or SA sample. This method may be parallelized when the input consists of multiple strings by stepping backward from the end of each string in parallel.

### 4.2. pSAscan

A second baseline is to compute the SA directly from the input; for this computation, we use the external-memory algorithm pSAscan (Kärkkäinen et al., 2015), with available memory set to the memory required by bigbwt on the specific input; with the ratio of memory to input size obtained from bigbwt, pSAscan is the current state-of-the-art method to compute the SA. Once pSAscan has computed the full SA, the SA sample may be constructed by loading the input text *T* into memory, streaming the SA from the disk, and the application of Equation (1) to detect run boundaries. We denote this method of computing the SA sample by pSAscan+.

We compared the performance of all the methods on two data sets: (1) *Salmonella* genomes obtained from GenomeTrakr (Stevens et al., 2017); and (2) chromosome 19 haplotypes derived from the 1000 Genomes (1KG) Project phase 3 data (Auton et al., 2015). The *Salmonella* strains were downloaded from NCBI (NCBI BioProject PRJNA183844) and preprocessed by assembling each individual sample with IDBA-UD (Peng et al., 2012) and counting *k*-mers (*k* = 32) using KMC (Deorowicz et al., 2015). We modified IDBA by setting kMaxShortSequence to 1024 per public advice from the author to accommodate the longer paired end reads that modern sequencers produce. We sorted the full set of samples by the size of their *k*-mer counts and selected 1000 samples about the median. This avoids exceptionally short assemblies, which may be due to low read coverage, and exceptionally long assemblies, which may be due to contamination.

Next, we downloaded and preprocessed a collection of chromosome 19 haplotypes from 1KG Project. Chromosome 19 is 58 million base pairs in length and makes up around 1.9% of the total human genome sequence. Each sequence was derived using the bcftools consensus tool to combine the haplotype-specific (maternal or paternal) variant calls for an individual in the 1KG project with the chr19 sequence in the GRCH37 human reference, producing an FASTA record per sequence. All DNA characters besides A, C, G, Tm and N were removed from the sequences before construction.

We performed all experiments in this section on a machine with Intel(R) Xeon(R) CPU E5-2680 v2 @ 2.80 GHz and 324 GB RAM. We measured running time and peak memory footprint using/usr/bin/time −v, with peak memory footprint captured by the Maximum resident set size (kbytes) field and running time by the User Time and System Time field.

**FIG. 1.**
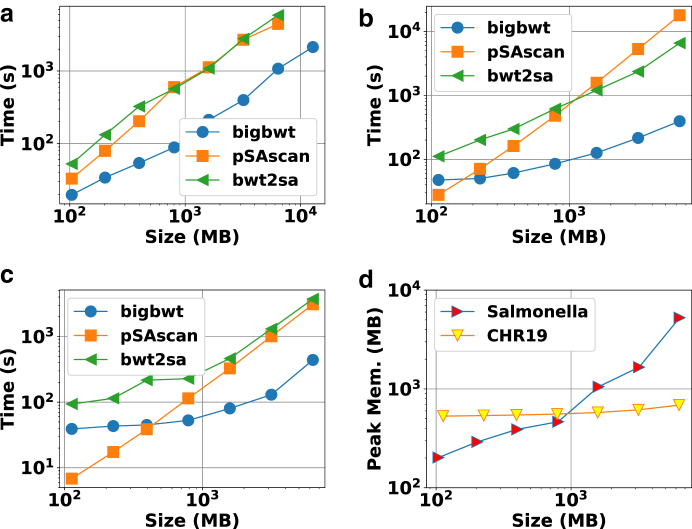
Runtime and peak memory usage for construction of full SA. **(a)** *Salmonella*, 1 thread. **(b)** chr19, 1 thread. **(c)** chr19, 16 threads. **(d)** Peak memory, bigbwt. SA, suffix array.

**FIG. 2.**
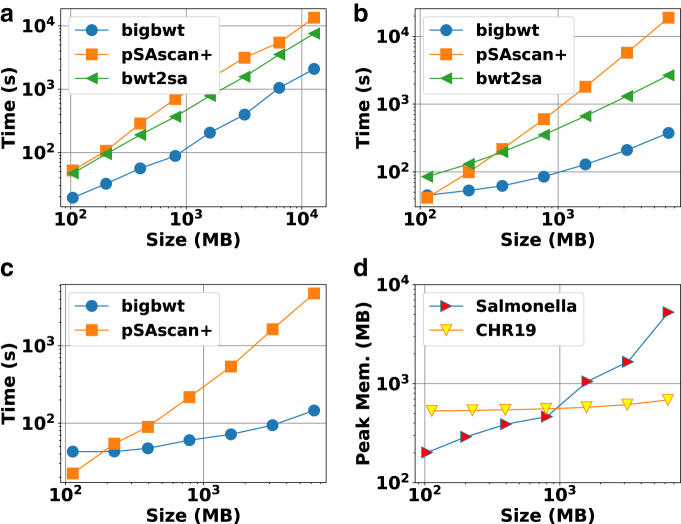
Runtime and peak memory usage for construction of SA sample. **(a)** *Salmonella*, 1 thread. **(b)** chr19, 1 thread. **(c)** chr19, 16 threads. **(d)** Peak memory, bigbwt.

We witnessed that the running time of each method to construct the full SA is shown in Figure 1a–c. On both the *Salmonella* and chr19 data sets, bigbwt ran the fastest, often by more than an order of magnitude. In Figure 1d, we show the peak memory usage of bigbwt as a function of input size. Empirically, the peak memory usage was sublinear in input size, especially on the chr19 data, which exhibited a high degree of repetition. Despite the higher diversity of the *Salmonella* genomes, bigbwt remained space-efficient and the fastest method for construction of the full SA. Furthermore, we found qualitatively similar results for construction of the SA sample, shown in Figure 2. Similar to the results on full SA construction, bigbwt outperformed both baseline methods and exhibited sublinear memory scaling on both types of databases.

## 5. Comparison with BOWTIE and CHIC

We studied how *r*-index scales to repetitive texts consisting of many similar genomic sequences, comparing it with Bowtie (version 1.2.2) (Langmead et al., 2008), a traditional FM-index-based aligner, and CHIC (Valenzuela and Mäkinen, 2017), a Hybrid Index that uses LZ compression to scale to repetitive texts. We measured indexing memory footprint, indexing time, index size, and locate query time.

We ran Bowtie with the −v 0 and –norc options; −v 0 disables approximate matching, while –norc causes Bowtie (like *r*-index) to perform the locate query with respect to the query sequence only and not its reverse complement.

CHIC parses the text with an LZ-like compression algorithm, storing the resulting phrases in a kernel string that can be indexed and aligned to with a standard aligner. Kernel-string alignments are transformed back to the original text coordinates using range-finding data structures. CHIC parameters include the following: which LZ parsing algorithm to use, the text prefix length from which phrases in the parse can be sourced (if a relative LZ algorithm is specified), the kernel-string indexing method, and the maximum length of the query patterns. We used the RLZ parsing method and the FM-index method for indexing the kernel string in all our experiments. For the prefix length, we tried both 10% and 30% of the text length. For the maximum query length, we tried both 100 and 250 bp, these being realistic second-generation sequencing read lengths. We refer to each parameter combination as “CHIC_*X*p_*Y*b,” where *X* is the prefix-length percentage and *Y* is the maximum query length.

### 5.1. Indexing chromosome 19s

We performed our experiments on collections of one or more haplotypes of chromosome 19. These haplotypes were obtained from the 1KG Project in the manner described in the previous section. We used 10 collections of chromosome 19 haplotypes, containing 1, 2, 10, 30, 50, 100, 250, 500, and 1000, 1250, 1500, and 2000 sequences, respectively. Each collection is a superset of the previous. Again, all DNA characters besides A, C, G, T, and N were removed from the sequences before construction. All experiments in this section were run on an Intel Xeon system with an E5-2680 v3 CPU clocked at 2.50 GHz and 512 GB memory. We measured running time and peak memory footprint as described in the previous section.

**FIG. 3.**
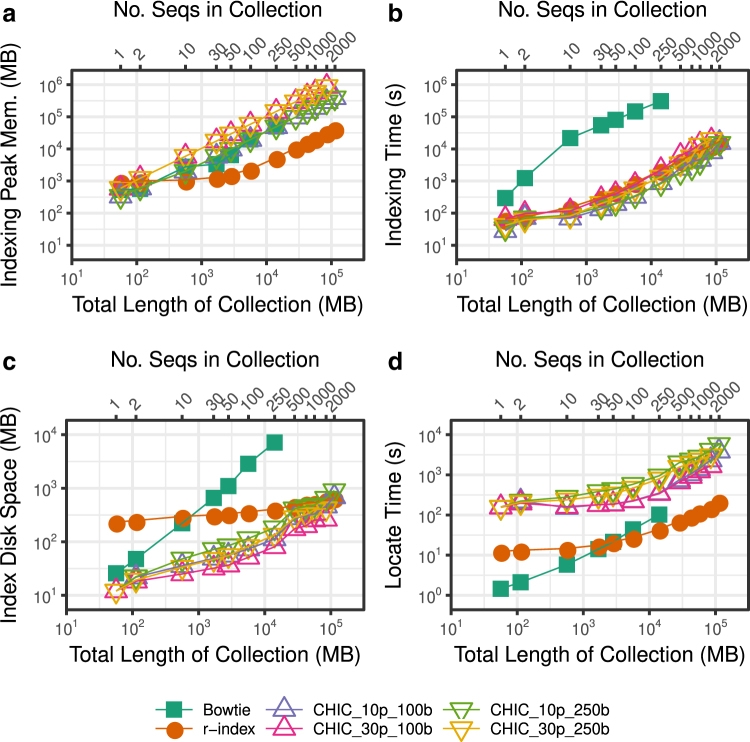
Scalability of r-index, Bowtie, and CHIC (RLZ compressed, FM-index kernel) against chr19 haplotype collection size and total sequence length (megabases) with respect to index construction time (seconds) **(a)**, index construction peak memory (megabytes) **(b)**, index disk space (megabytes) **(c)**, and locate time (seconds) of 100,000 one hundred base pair queries **(d)**. Four different CHIC indexes were used, using different combinations of prefix size and maximum query length, each labeled as *CHIC*_(prefix size)*p*_(max query length).

First, we constructed *r*-index, Bowtie, and CHIC indexes for successively larger chromosome 19 collections (Fig. 3a, b). *r*-Index uses the least indexing memory for collections of 10 chromosomes and larger. At 250 chr19s, the *r*-index procedure takes about 2% of the time and 6% of the peak memory of Bowtie's procedure.

While CHIC's peak memory is also much higher than *r*-index's at 10 sequences and above, CHIC tends to construct indexes faster, especially when using a prefix length of 10% of the text. At 2000 sequences, CHIC_10p_100b takes about 64% of the time, but 920% of the memory of *r*-index. Bowtie is drastically slower to index than either CHIC or *r*-index, especially for larger collections. Due to memory exhaustion, Bowtie fails to index collections of more than 250 sequences and 2 of the CHIC modes (those using a 30% prefix) fail for collections of more than 1500 sequences.

Next, we compared the disk footprint of the index files produced by all three methods (Fig. 3c). *r*-Index currently stores only the forward strand of the sequence. Bowtie, on the contrary, stores both the forward sequence and its reverse as needed by its double-indexing heuristic (Langmead et al., 2008). Since the heuristic is relevant only for approximate matching, we omit the reverse sequence in these size comparisons. We also omit the 2-bit encoding of the original text (in the *.3.ebwt and *.4.ebwt files) as these too are used only for approximate matching. Specifically, the Bowtie index size was calculated by adding the sizes of the forward *.1.ebwt and *.2.ebwt files, which contain the BWT, SA sample, and auxiliary data structures for the forward sequence. CHIC stores the forward strand of the kernel string, along with range-finding data structures. These consist of the files ending with *.P512_GC4_kernel_text.MAN.kernel_index (the kernel index), and *.book_keeping, *.is_literal, *.limits, *.limits_kernel, *.ptr, *.rmq, *.sparse_sample_limits_kernel, *.sparseX, *.variables and *.x (the range-finding data structures).

An *r*-index is considerably larger than a Bowtie or CHIC index for smaller collections. However, it grows at a slower rate than any of the other indexes, becoming smaller than Bowtie at 30 sequences and smaller than CHIC_10p_250b at 1500 sequences. The *r*-index incurs more overhead for smaller collections because SA sample density depends on the ratio *n*/*r*. When the collection is small, *n*/*r* is small leading to a denser SA sample than the 1-in-32 rate used by Bowtie. The CHIC index stays small by indexing only the kernel string, which is smaller than the text. Like Bowtie, the FM-index kernel samples a constant fraction of SA elements. Finally, CHIC's range-query data structures are typically smaller than the kernel index. At 250 sequences, the *r*-index takes 6% the space of the Bowtie index and 509% the space of the CHIC_30p_100b index (the smallest CHIC index at this point). At 1500 sequences, the CHIC_30p_100b index takes 45% the space of *r*-index.

We then compared the speed of the locate query for the *r*-index, Bowtie, and Compressed Hybrid Index of (Repetitive) Collections (Aligner) [CHIC]. We extracted 100,000 one hundred-character substrings from the chr19 collection of size 1, which is also contained in all of the larger collections. We queried these against each of the indexes constructed. We aimed to measure the speed of locating *all* occurrences of each pattern, because in repetitive indexes, the number of occurrences for one pattern is on average the number of sequences in the collection, but it could also exceed that number due to multimapping within a sequence. Since the source of the substrings is present in all the collections, every query will match at least once. As seen in Figure 3d, the *r*-index locate time is faster than that of Bowtie after 50 sequences, and it is consistently at least 10 × faster than any of the CHIC modes.

## 6. Indexing Whole Human Genomes

Lastly, we used *r*-index to index many human genomes at once. We repeated our measurements for successively larger collections of (concatenated) genomes. Thus, we first evaluated a series of haplotypes extracted from the 1KG Project (Auton et al., 2015) phase 3 callset (1KG). These collections ranged from 1 up to 10 genomes. As the first genome, we selected the GRCh37 reference itself. For the remaining nine, we used bcftools consensus to insert single nucleotide variations (SNVs) and other variants called by the 1KG Project for a single haplotype into the GRCh37 reference.

Second, we evaluated a series of whole human genome assemblies from six different long-read assembly projects (“LRA”). We selected GRCh37 reference as the first genome, so that the first data point would coincide with that of the previous series. We then added long-read assemblies from a Chinese genome assembly project (Shi et al., 2016), a Korean genome assembly project (Seo et al., 2016), a project to assemble the well-studied NA12878 individual (Jain et al., 2018), a hydatidiform mole (known as CHM1) assembly project (Steinberg et al., 2014), and the Celera human genome project (Levy et al., 2007). Compared with the series with only 1KG Project individuals, this series allowed us to measure scaling while capturing a wider range of genetic variations between humans. This is important since *de novo* human assembly projects regularly produce assemblies that differ from the human genome reference by megabases of sequence (12 megabases in the case of the Chinese assembly; Shi et al., 2016), likely due to prevalent but hard-to-profile large-scale structural variation. Such variation was not comprehensively profiled in the 1KG Project, which relied on short reads.

The 1KG and LRA series were evaluated twice, once on the forward genome sequences and once on both the forward- and reverse-complement sequences. This accounts for the fact that different *de novo* assemblies make different decisions about how to orient contigs. The *r*-index method achieves compression only with respect to the forward-oriented haplotypes of the sequences indexed. That is, if two contigs are reverse complements of each other but otherwise identical, the *r*-index achieves less compression than if their orientations matched. A more practical approach would be to index both forward- and reverse-complement sequences, as Bowtie 2 (Langmead and Salzberg, 2012) and BWA (Li, 2013) do.

**FIG. 4.**
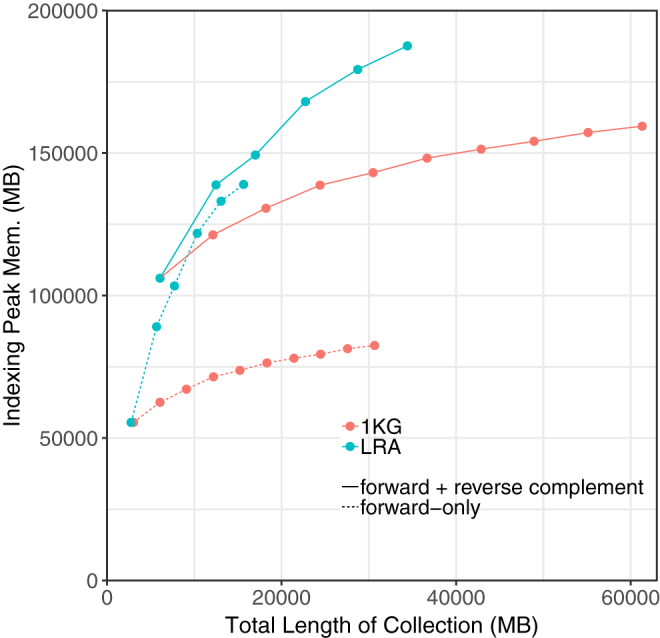
Peak index-building memory for *r*-index when indexing successively larger collections of 1KG individuals and whole-genome long-read assemblies (LRA). 1KG, 1000 genomes.

We measured the peak memory footprint when indexing these collections (Fig. 4). We ran these experiments on an Intel(R) Xeon(R) CPU E5-2650 v4 @ 2.20 GHz system with 256 GB memory. Memory footprints for LRA grew more quickly than those for 1KG. This was expected due to the greater genetic diversity captured in the assemblies. This may also be due, in part, to the presence of sequencing errors in the long-read assembles; long-read technologies are more prone to indel errors than short-read technologies, for example, and some may survive in the assemblies. Also as expected, memory footprints for the LRA series that included both forward- and reverse-complement sequences grew more slowly than when just the forward sequence was included. This is due to sequences that differ only (or primarily) in their orientation between assemblies. All series exhibit sublinear trends, highlighting the efficacy of *r*-index compression even when indexing genetically diverse whole-genome assemblies. Indexing the forward- and reverse-complement strands of 10 1KG individuals took about 6 hours and 20 minutes and the final index size was 36 GB.

We also measured lengths and *n*/*r* ratios for each collection of whole genomes (Table 1). Consistent with the memory-scaling results, we see that the *n*/*r* ratios are somewhat lower for the LRA series than for the 1KG series, likely due to greater genetic diversity in the assemblies.

**Table 1.** Sequence Length and *n*/*r* Statistic with Respect to Number of Whole Genomes for the First 6 Collections in the 1000 Genomes and Long-Read Assembly Series

No. of genomes	Sequence			
	Length (MB)		n/r	
	1KG	LRA	1KG	LRA
1	6072	6072	1.86	1.86
2	12,144	12,484	3.70	3.58
3	18,217	17,006	5.38	4.83
4	24,408	22,739	7.13	6.25
5	30,480	28,732	8.87	7.80
6	36,671	34,420	10.63	9.28

1KG, 1000 genomes; LRA, long-read assembly.

## 7. Conclusions and Future Work

We give an algorithm for building the SA and SA sample from the prefix-free parse of an input string *S*, which fully completes the practical challenge of building the index proposed by Gagie et al. (2018). This leads to a mechanism for building a complete index of large databases—which is the linchpin in developing practical means for pan-genomics short-read alignment. We apply our method for indexing partial and whole human genomes, and show that it scales better than Bowtie with respect to both memory and time. This allows for an index to be constructed for large collections of chromosome 19s (500 or more); a task that is out of reach of Bowtie, causing it to exhaust memory even with a budget of 512 GB. Our method produces indexes in a smaller memory footprint than a Hybrid Index-based method (CHIC; Valenzuela and Mäkinen, 2017) while providing much faster locate time.

Although this work opens the door to indexing large collections of genomes, it also highlights problems needing further investigation. A major question is how this work can be adapted to work on large sets of sequence reads. This problem not only requires the construction of the *r*-index but also adapting and incorporating efficient means (Bannai et al., 2018) to update the index as new data sets become available. Moreover, the use of many reference sequences complicates the task of a read aligner performing approximate matching. In the future, it will be important to explore both techniques, such as *r*-index, that can facilitate the seed-finding phase of approximate matching, and also techniques—perhaps such as those proposed in entropy-scaling search (Yu et al., 2015)—that can facilitate the gapped extension phase.

## Notes

^*^ With the SA sample of Gagie et al. (2018), this index is termed the *r*-index.

^†^ Given a sequence (string) *S*[1, *n*] over an alphabet Σ = {1, …, *σ*}, a character *c* ∈Σ, and an integer *i*, ${rank}_{c}\left(S,i\right)$ is the number of times that *c* appears in *S*[1, *i*].

^‡^ *Sampled* means that only some fractions of entries of the suffix array are stored.

^§^ For technical reasons, the string *S* must terminate with *w* copies of lexicographically least $ symbol.
